# The Effect of Phototherapy on Cancer Predisposition Genes of Diabetic and Normal Human Skin Fibroblasts

**DOI:** 10.1155/2017/7604861

**Published:** 2017-03-12

**Authors:** Pongsathorn Chotikasemsri, Boonsin Tangtrakulwanich, Surasak Sangkhathat

**Affiliations:** ^1^Biomedical Engineering Institute, Faculty of Medicine, Prince of Songkla University, Hat Yai, Songkhla, Thailand; ^2^Department of Orthopedic Surgery and Physical Medicine, Faculty of Medicine, Prince of Songkla University, Hat Yai, Songkhla, Thailand; ^3^Department of Surgery, Faculty of Medicine, Prince of Songkla University, Hat Yai, Songkhla, Thailand

## Abstract

The purpose of this study was to investigate whether LED light at different wavelengths affects the expression profile of 143 cancer predisposition genes in both diabetic and normal human fibroblasts. In this study, both diabetic and normal fibroblast cell lines were cultured and irradiated with red (635 nm), green (520 nm), and blue (465 nm) LED light for 10 minutes at 0.67 J/cm^2^ each. After that, mRNA from all cell lines was extracted for microarray analysis. We found that green light activates EPHB2, KIT, ANTXR2, ESCO2, MSR1, EXT1, TSC1, KIT, NF1, BUB1B, FANCD2, EPCAM, FANCD2, NF, DIS3L2, and RET in normal fibroblast cells, while blue and red light can upregulate RUNX1, PDGFRA, EHBP1, GPC3, AXIN2, KDR, GLMN, MSMB, EPHB2, MSR1, KIT, FANCD2, BMPR1A, BUB1B, PDE11A, and RET. Therefore, genetic screening before phototherapy treatment may be required.

## 1. Introduction

Nowadays, there are a number of light-assisted treatments for medical application, such as UV-B treatment for psoriasis, red light for acne vulgaris, daylight spectrum for seasonal affected disorder, Levulan blue light for skin psoriasis or jaundice, and GreenLight Laser PVP (Photoselective Vaporization of the Prostate) for benign prostatic hyperplasia. However, there have been problems noted with light treatments; for example, Valejo Coelho and Apetato [1] did a study on adverse effects of phototherapy which could lead to certain skin cancers. In addition, other studies from Wickremasinghe et al. [2] and Frazier et al. [3] have noted a significant increased risk of cancer in infants after neonatal phototherapy.

There are 143 well-known hereditary cancer predisposition genes (Table 1) that have been documented for genetic analysis. The mutation of these genes serves as markers of cancer cells after pathological and biopsy analysis. Therefore, the question arises; if any patients who have been previously diagnosed with mutation of such genes need phototherapy or light-assisted treatments, will there be any effect on these mutant gene expression levels? As a number of wavelengths have been used, how certain wavelengths affect the whole genes and their expression has not been clarified.

This study aimed to evaluate the changes of expression levels of cancer predisposition genes after irradiation with red (635 nm), green (520 nm), and blue (465 nm) LED light by using microarray and cell culture techniques.

## 2. Materials and Methods

### 2.1. LED Light Source

An apparatus equipped with blue (465 nm), green (520 nm), and red (635 nm) light emitting diodes with a 1 ampere power supply was constructed for this experiment. Calibrating analysis of the light's emission spectra and power was provided by a monochrome meter and a power meter from the Department of Physics, Prince of Songkla University. The LED diode array (15 cm by 15 cm), assembled by a technician from the Department of Electrical Engineering, Prince of Songkla University, consisted of three different wavelength array panels that could be placed over and perfectly cover a 10 cm cell culture plate. In this array, the blue LED emitted light between 440 and 500 nm with peak emission at 465 nm, the green LED emitted light between 495 and 575 nm with peak emission at 520 nm, and the red LED emitted light between 610 and 660 nm with peak emission at 635 nm.

### 2.2. Cell Culture Assay

Both the type 2 diabetic fibroblast cell line from a donor diagnosed with type II diabetes (untransformed, Caucasian, MODY, aged 23 years, Cat. #AG06083) and healthy normal fibroblast cell line (male, leg skin, untransformed, Caucasian, Cat. #GM03440) were obtained from the Coriell Institute for Medical Research and cultured at 37°C 5% CO_2_ in DMEM (supplemented with 10% FBS and 100 *μ*g/ml streptomycin Penn Strep) from GIBCO in 10 cm plates with 2,000,000 cells of initial seeding density. During the entire experiment, these cells were maintained and used in not more than five passages. The type 2 diabetic fibroblast cell line and healthy normal fibroblast cell line were cultured at the regular normal glucose level under the assumption that they were from glycemic well-controlled patients. The type 2 diabetic fibroblast cell line and healthy normal fibroblast cell line served as treated groups and control groups, respectively.

### 2.3. LED Irradiation Assay and mRNA Isolation

Before LED-light exposure, the cells were cultured until 90% confluence and a number of artificial wounds were randomly created with a 1 ml plastic pipette tip in order to reduce bias of the result and simulate the real wounded shape. Then, in the treatment groups, 3 groups each of diabetic fibroblasts and normal fibroblast cells were exposed to the light at 0.67 J/cm^2^ for 10 minutes at different wavelengths. According to our preliminary experiment, the light irradiating energy at 0.67 J/cm^2^ was the optimum level for this in vitro experiment. One of the diabetic fibroblasts and one healthy normal fibroblast received no light exposure to serve as controls. The details of the treated groups are shown in Table 2. The energy power of the light that the cells received was carefully calibrated by an optical power meter. The irradiation distance from the LED diode array to the media surface was 10 cm. The apparatus had been placed in a laminar flow (ESCO class II), and a cooling airflow fan constantly maintained a constant temperature during the light exposure. After that, all cells were suddenly trypsinized and their mRNA extracted. All extractions were performed with a GeneJet RNA purification kit from Thermo Scientific. Each condition was pentaplicated for reliable and valid results.

### 2.4. mRNA Microarray Assay

All mRNA samples were shipped to a certified Agilent microarray service in India and the purity of the mRNA samples was tested by Bioanalyzer with an RNA integrity number (RIN) higher than 8.0 before carrying on to hybridize with the microarray chips. SurePrint G3 human gene expression 8x60k V2 chips were used for this experiment.

### 2.5. Statistical and Microarray Data Analysis

All raw data were analyzed by GeneSpring 13. All gene expression profiles with a 2-fold or greater difference were selected and followed by differential gene expression analysis (fold change analysis), gene ontology analysis, and pathway analysis. For differential gene expression analysis (fold change analysis), it was calculated by the absolute ratio of normalized intensities (no log scale) between the average intensities of the samples grouped. For gene ontology (GO) analysis, it was an analysis to observe how significantly such genes can affect certain function of a cell. This analysis was calculated by using Benjamini-Yekutieli procedure with corrected *p* values. Finally, for pathway analysis, curated pathways from the WikiPathways.org were imported to GeneSpring 13 software in order to visualize every pathway that such genes had been affected. The cut-off level for all analyses was a *p* value less than 0.05. The raw data were also submitted to the NCBI database for other GEO researchers to use.

## 3. Results

### 3.1. Differential Gene Expression

**Figure 1:**
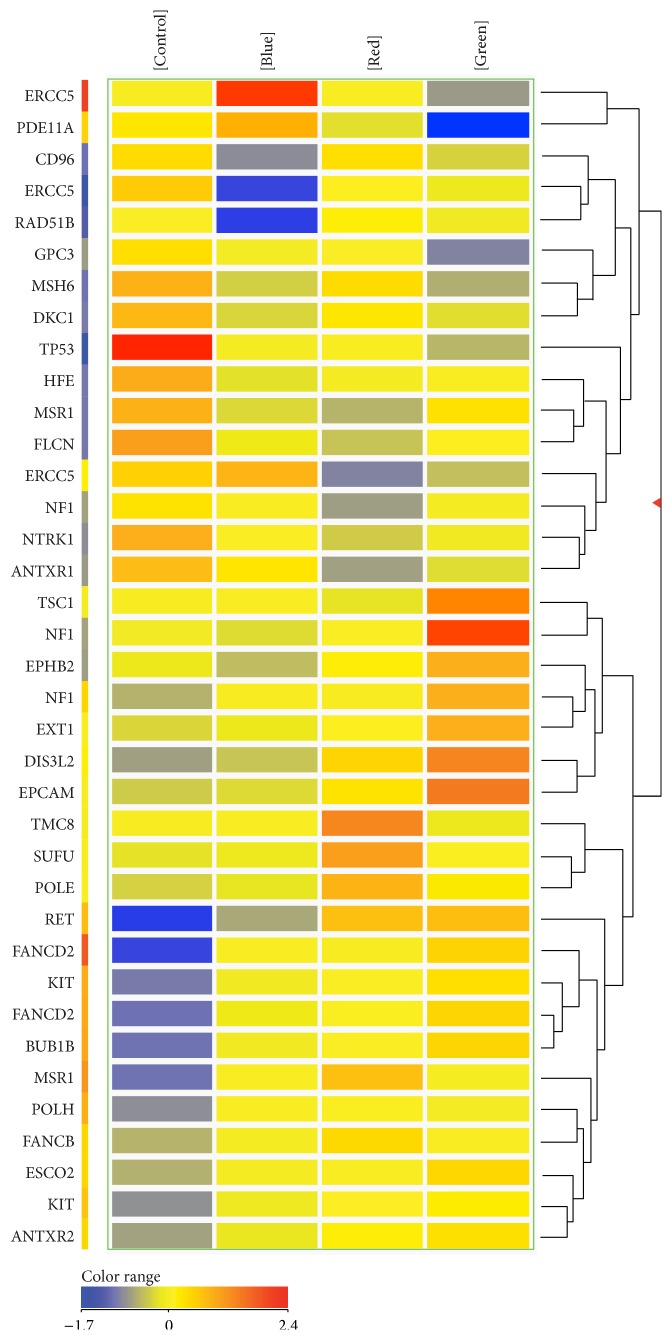
37 out of 143 genes were significantly up- or downregulated with more than 2-fold differences after irradiation with red, green, or blue light in normal skin fibroblast cells (*p* < 0.05).

**Figure 2:**
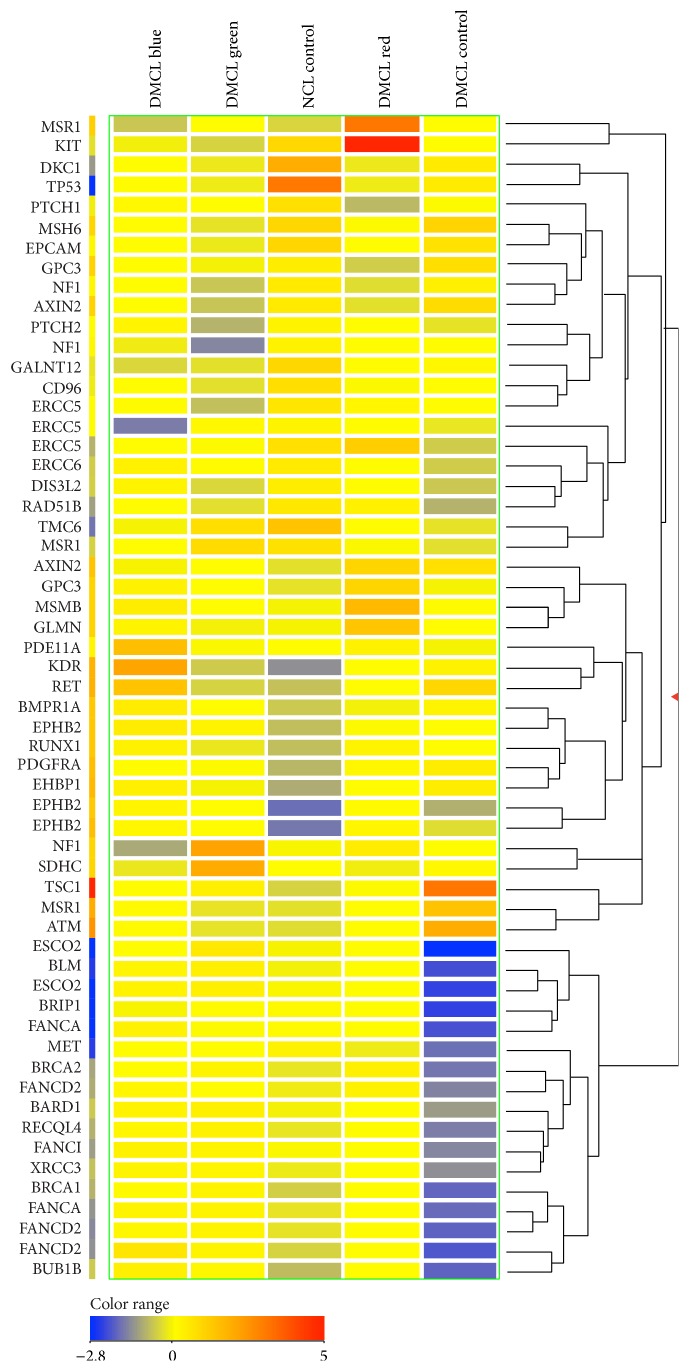
58 out of 143 genes were significantly up- and downregulated for 2-fold differences after irradiation with red, green, or blue light in diabetic fibroblast cells (*p* < 0.05).

After the mRNA samples of the fibroblast cells were analyzed by GeneSpring 13, all results were categorized and compared as shown in Figures 1 and 2. Total gene expression profiles and raw data can be accessed through the GEO database of the NCBI at GSE78017 and GSE78018. Only 143 predisposition cancer genes were selected and further analyzed, comparing them with untreated normal human fibroblasts (control group).  Figure 1 shows 37 genes from normal human fibroblast cells of each treatment group which were significantly 2-fold or more up or down (*p* < 0.05). In the NCL blue group, BUB1B, MSR1, FANCD2, and ERCC5 were significantly upregulated, while DKC1, HFE, MSR1, FLCN, MSH6, CD96, RAD51B, ERCC5, and TP53 genes were significantly downregulated. In the NCL green group, the EPHB2, KIT, ANTXR2, ESCO2, MSR1, EXT1, TSC1, BUB1B, EPCAM, FANCD2, NF1, DIS3L2, and RET genes were significantly expressed at higher levels, while 6 genes, DKC1, ERCC5, GPC3, MSH6, PDE11A, and TP53, showed significantly lower expression. In the NCL red group, FANCB, POLH, KIT, POLE, BUB1B, DIS3L2, FANCD2, SUFU, TMC8, MSR1, and RET had significantly high expression, while NF1, NTRK1, MSR1, ANTXR1, ERCC5, FLCN, and TP53 were expressed at significantly lower levels.

Figure 2 shows 54 genes from diabetic human fibroblast cells of each treatment group which were significantly over- or downexpressed 2-fold or more (*p* < 0.05). In the DMCL blue group, RUNX1, FANCD2, BMPR1A, BUB1B, EPHB2, EHBP1, PDE11A, RET, and KDR were significantly upregulated, while NF1, KIT, GALNT12, TMC6, ERCC5, DKC1, and TP53 were significantly downregulated. In the DMCL green group, only EPHB2, SDHC, and NF1 genes were significantly expressed at higher levels, while 12 genes, CD96, PTCH2, AXIN2, NF1, EPCAM, MSH6, GALNT12, ERCC5, KIT, NF1, DKC1, and TP53, had significantly lower expression. And in the DMCL red group, RUNX1, PDGFRA, EHBP1, GPC3, AXIN2, KDR, GLMN, MSMB, EPHB2, MSR1, and KIT had significantly high expression, and 5 genes, GPC3, TMC6, PTCH1, DKC1, and TP53 genes, were expressed at significantly lower levels.

### 3.2. Gene Ontology Analysis of the Blue Light Irradiation Group

For the groups which were irradiated with blue light and compared with NCL, only 4 genes, BUB1B, MSR1, FANCD2, and ERCC5, were upregulated after blue light irradiation in normal fibroblast cells. However, there were no significant changes to gene ontology. In contrast, 9 genes were detected with significantly lower expression, DKC, HFE, MSR, FLCN, MSH6, CD96, RAD51B, ERCC5, and TP53. These suppressed genes are known to have a direct impact on lowering DNA repair activity during the replication fork process.

In addition, in the DMCL blue group, the 9 genes shown in Table 3 (RUNX1, FANCD2, BMPR1A, BUB1B, EPHB2, EHBP1, PDE11A, RET, and KDR) were highly activated and all of them can significantly affect embryonic hemopoiesis, transferase activities, and protein phosphorylation. On the other hand, 7 genes were clearly observed to have lower expression, NF, KIT, GALNT12, TMC6, ERCC5, DKC, and TP53. These genes were significantly downregulated, and downregulation of these genes has a negative effect on the regulation of neuroblast proliferation.

### 3.3. Gene Ontology Analysis of Green Light Irradiation

Sixteen genes were upregulated in the NCL green group after green light irradiation in normal fibroblast cells. This set of genes could significantly affect transmembrane receptor protein tyrosine kinase activities. In contrast, 6 genes (DKC1, ERCC5, GPC3, MSH6, PDE11A, and TP53) were detected with significantly lower expression (Table 3). These suppressed genes directly affected structure-specific DNA secondary structure binding for DNA repair, replication, and DNA metabolic process.

In addition, in the DMCL green group, only 3 genes, EPHB2, SDHC, and NF, were highly activated; all of these genes are significantly involved in the regulation of neuronal synaptic plasticity. On the other hand, the other 12 genes, CD96, PTCH2, AXIN2, NF1, EPCAM, MSH6, GALNT12, ERCC5, KIT, NF1, DKC1, and TP53, had lower expression (Table 3). These 12 genes were significantly downregulated which affected the cellular immune response, DNA metabolic process, the negative regulation of neuroblast proliferation, response to light stimulus, epithelium development, and maintenance of DNA repeat elements.

### 3.4. Gene Ontology Analysis of Red Light Irradiation

In the NCL red group in which the cell lines were irradiated with red light and compared with NCL, there were no significant positive changes to gene ontology (12 genes, Table 3). In contrast, 7 genes, NF1, NTRK1, MSR1, ANTXR1, ERCC5, FLCN, and TP53, showed significantly lower expression. These suppressed genes are known to have a direct impact on RAS protein signal transduction, the negative regulation of neuroblast proliferation, sympathetic nervous system development, and the positive regulation of adenylate cyclase activities.

In addition, in the DMCL red group, 12 genes (RUNX1, PDGFRA, EHBP1, GPC3, AXIN2, KDR, GLMN, MSMB, EPHB2, MSR1, and KIT, Table 3) were upregulated in normal fibroblast cells. These highly active genes directly affected protein kinase activities, vascular endothelial growth factor binding, ovarian follicle development, and hematopoietic progenitor cell differentiation (immune system development). On the other hand, only 5 genes in this group were clearly observed to have lower expression, GPC3, TMC6, PTCH1, DKC1, and TP53. This set of genes is known to have a direct effect on the process of cell proliferation involved in metanephros development of the kidney.

## 4. Discussion

We found that phototherapy can significantly affect the expression of certain brain, lung, breast, ovarian, and prostate cancer “predisposition genes” in nondiabetic patients and also increase the risk of juvenile intestinal polyposis, hemangioma, multiple endocrine neoplasia, endometrial cancer, and chronic myeloid leukemia in diabetic patients.

Green light irradiation may significantly increase the chances for a number of cancers in nondiabetic patients (Table 4). For example, irradiated patients are at increased risk of developing hyaline fibromatosis, neurofibromatosis, brain cancer, lung cancer, prostate cancer, breast cancer, Wilms tumor, multiple endocrine neoplasia, Barrett's esophagus/esophageal adenocarcinoma, and endometrial cancer in patients with an existing gastrointestinal stromal tumor. In contrast, the green light irradiation could develop only a few types of cancers, such as brain cancer, prostate cancer, neurofibromatosis, and paragangliomas in diabetic patients (Table 5). Thus, patients who require prostate surgery with a green light laser should be carefully checked to see whether they have mutated predisposition genes before their surgery. In addition, using excessive blue light for skin psoriasis and jaundice treatment may increase the occurrences of particular cancers in newborns who have been genetically diagnosed with these mutated genes.

For red light irradiation, prostate cancer, breast cancer, brain cancer, lung cancer, Wilms' tumor, Fanconi anemia, Barrett's esophagus/esophageal adenocarcinoma, xeroderma pigmentosum, medulloblastoma, epidermodysplasia verruciformis, colorectal cancer, and endometrial cancer in gastrointestinal stromal tumor could develop in nondiabetic patients (Table 4). On the other hand, for diabetic patients considered for red light irradiation, the high expression of such genes may increase the rates of colorectal cancer, prostate cancer, Barrett's esophagus/esophageal adenocarcinoma, glomangioma, Wilms' tumor, hemangioma, and myeloid malignancy (Table 5). Hence, a red light phototherapy for acne vulgaris and other cosmetic purposes could be a potential threat that the doctors and diabetic patients should be aware of.

In short, according to the results of this experiment, any wavelength can either increase or lower expression of particular mutated predisposition cancer genes. Therefore, awareness of potential side effects should be considered before any proposed laser operation. In addition, one can take advantage of this phototherapy to either be aware of the side effects on how light can induce the expression of certain predisposition cancer genes or suppress certain mutated genes to reduce causes of cancers. In contrast, one may take advantage of how phototherapy downregulates or suppresses the expression of certain predisposition genes which may reduce the progression of cancer cells to develop further. For example, the expression of mutated TP53 gene, a cancer marker gene causing Li-Fraumeni syndrome, breast cancer, soft tissue sarcoma, osteosarcoma (bone cancer), leukemia, brain tumors, and adrenocortical carcinoma, can be reduced by irradiating with blue, green, or red light. In addition, both ERCC5 and TP53 genes were found with significantly lower expression in all treated groups. These genes work as tumor suppressor genes; therefore, intense phototherapy could definitely decrease the DNA repair function. Moreover, lower expression of DKC1 was also found, which is important as DKC1 is able to control the length of telomerases after the cell cycle. Eventually, either more abnormal cells would be produced or more apoptosis could occur. These hypotheses are supported by previous studies from Płonka et al. [4] and Acedo and Zawacka-Pankau [5]. These studies found that the expression of the P53 (also called TP53) gene was lower after phototherapy, which led to the initiation of the apoptosis process.

In addition, for patients with neurofibromatosis type 1 (a mutated NF1 gene), intense pulsed-radio frequency (IPL-RF) in combination with topical application of vitamin D3 ointment is one of the current treatments. What if we use red light to suppress the expression of this mutated NF1? Could it improve the quality of their lives? Our study suggests that red light irradiation could significantly reduce the expression of the NF1 gene in nondiabetic patients and blue light irradiation significantly lowers the expression of NF1 gene in diabetic patients.

Therefore, gene screening for these 143 predisposition genes should be implemented before any phototherapy treatment and further investigations are strongly recommended to explore the benefits of phototherapy for cancer treatment.

## 5. Conclusion

Phototherapy increases the expression of cancer predisposing genes in both normal and diabetic cell lines. Green light activates EPHB2, KIT, ANTXR2, ESCO2, MSR1, EXT1, TSC1, KIT, NF1, BUB1B, FANCD2, EPCAM, FANCD2, NF, DIS3L2, and RET in normal fibroblast cells, whereas blue and red light can upregulate RUNX1, PDGFRA, EHBP1, GPC3, AXIN2, KDR, GLMN, MSMB, EPHB2, MSR1, KIT, FANCD2, BMPR1A, BUB1B, PDE11A, and RET. Genetic screening before applying phototherapy may be warranted.

## Figures and Tables

**Table 1:** There are 143 predisposition genes that have been widely used for SNP detection of various types of cancer.

AIP	APC	BAP1	BRCA2
AKT1	ASCC1	BARD1	BRIP1
ALK	ATM	BLM	BUB1B
ANTXR1	ATR	BMPR1A	CD96
ANTXR2	AXIN2	BRCA1	CDC73
CDH1	FANCI	MUTYH	RUNX1
CDK4	FANCL	NBN	SBDS
CDKN1B	FANCM	NDUFA13	SDHA
CDKN2A	FH	NF1	SDHAF2
CHEK1	FLCN	NF2	SDHB
CHEK2	GALNT12	NTRK1	SDHC
CYLD	GATA2	PALB2	SDHD
CYP21A2	GL13	PALLD	SLX4
DDB2	GLMN	PDE11A	SMAD4
DICER1	GPC3	PDGFRA	SMARCA4
DIS3L2	HFE	PIK3CA	SMARCB1
DKC1	HRAS	PMS2	STK11
EHBP1	KDR	POLD1	SUFU
EPCAM	KIF1B	POLE	TERT
EPHB2	KIT	POLH	TGFBR1
ERCC2	KLHDC8B	POU6F2	TINF2
ERCC3	LIG4	PRKAR1A	TMC6
ERCC4	LYST	PTCH1	TMC8
ERCC5	MAX	PTCH2	TMEM127
ERCC6	MC1R	PTEN	TP53
ESCO2	MEN1	RAD50	TSC1
EXT1	MET	RAD51B	TSC2
EXT2	MITE	RAD51C	UROD
FAH	MLH1	RAD51D	VHL
FANCA	MLH3	RB1	WAS
FANCB	MRE11A	RECQL4	WRN
FANCC	MSH2	RET	WT1
FANCD2	MSH6	RHBDF2	XPA
FANCE	MSMB	RNASEL	XPC
FANCF	MSR1	RSPO1	XRCC3
FANCG	MTAP	RTEL1	

**Table 2:** Assigned abbreviations for each condition.

Untreated	Treated with red light	Treated with green light	Treated with blue light
NCL control	NCL red	NCL green	NCL blue
DMCL control	DMCL red	DMCL green	DMCL blue

**Table 3:** Summary of genes that were affected from each treatment (*p* < 0.05).

		Blue		Green		Red	
NCL	Up	BUB1B	FANCD2	EPHB2	BUB1B	FANCB	FANCD2
		MSR1	ERCC5	KIT	EPCAM	POLH	SUFU
				ANTXR2	FANCD2	KIT	TMC8
				ESCO2	NF1	POLE	MSR1
				MSR1	DIS3L2	BUB1B	RET
				EXT1	RET	DIS3L2	
				TSC1			
						
	Down	DKC1	CD96	DKC1	MSH6	NF1	ERCC5
		HFE	RAD51B	ERCC5	PDE11A	NTRK1	FLCN
		MSR1	ERCC5	GPC3	TP53	MSR1	TP53
		FLCN	TP53			ANTXR1	
		MSH6					

DMCL	Up	RUNX1	PDE11A	EPHB2		RUNX1	GLMN
		FANCD2	RET	SDHC		PDGFRA	MSMB
		BMPR1A	KDR	NF1		EHBP1	EPHB2
		BUB1B				GPC3	MSR1
		EPHB2				AXIN2	KIT
		EHBP1				KDR	
						
	Down	NF1	ERCC5	CD96	GALNT12	GPC3	DKC1
		KIT	DKC1	PTCH2	ERCC5	TMC6	TP53
		GALNT12	TP53	AXIN2	KIT	PTCH1	
		TMC6		NF1	NF1		
				EPCAM	DKC1		
				MSH6	TP53		

**Table 4:** Cancer-related predisposition genes that were overexpressed after blue, green, or red light irradiation in normal healthy fibroblast cells (nondiabetic patients) (*p* < 0.05).

Light condition	Overexpressed genes after irradiation	Possible cancers affected
Blue	BUB1B	Brain and lung cancer
	ERCC5	Ovarian cancer
	FANCD2	Breast cancer and Fanconi anemia
	MSR1	Prostate cancer and Barrett's esophagus/esophageal adenocarcinoma

Green	ANTXR2	Hyaline fibromatosis syndrome and hyalinosis, inherited systemically (its related pathways are infectious disease and uptake and actions of bacterial toxins)
	BUB1B	Brain and lung cancers
	DIS3L2	Perlman syndrome and Wilms' tumor susceptibility-5
	EPCAM	Endometrial cancer, biliary tract cancer, and skin cancer
	ESCO2	Roberts syndrome and SC phocomelia syndrome
	EPHB2	Prostate cancer/brain cancer susceptibility, somatic and prostate cancer
	EXT1	Chondrosarcoma and exostoses, multiple, type 1
	FANCD2	Breast cancer and Fanconi anemia
	KIT	Endometrial cancer as a gastrointestinal stromal tumor
	MSR1	Prostate cancer and Barrett's esophagus/esophageal adenocarcinoma
	NF1	Neurofibromatosis-Noonan syndrome and neurofibromatosis, type 1
	RET	Multiple endocrine neoplasia iia and medullary thyroid carcinoma, familial
	TSC1	Tuberous sclerosis-1 and lymphangioleiomyomatosis

Red	BUB1B	Brain and lung cancer
	DIS3L2	Perlman syndrome and Wilms' tumor susceptibility-5
	FANCB	Fanconi anemia, complementation group B, and Fanconi anemia, complementation group A
	FANCD2	Breast cancer and Fanconi anemia
	KIT	Endometrial cancer as a gastrointestinal stromal tumor
	MSR1	Prostate cancer and Barrett's esophagus/esophageal adenocarcinoma
	POLE	FILS syndrome and colorectal cancer 12
	POLH	Xeroderma pigmentosum, variant type and POHL-related xeroderma pigmentosum
	RET	Multiple endocrine neoplasia iia and medullary thyroid carcinoma, familial
	SUFU	Medulloblastoma and basal cell nevus syndrome
	TMC8	Epidermodysplasia verruciformis and superficial mycosis

**Table 5:** Cancer-related predisposition genes that were overexpressed after blue, green, or red light irradiation in diabetic fibroblast cells (*p* < 0.05).

Light condition	Overexpressed genes after irradiation	Possible cancers affected
Blue	BUB1B	Brain and lung cancers
	BMPR1A	Polyposis syndrome, hereditary mixed, 2 and polyposis, juvenile intestinal
	EHBP1	Prostate cancer, hereditary, 12 and prostate cancer
	EPHB2	Prostate cancer/brain cancer susceptibility, somatic and prostate cancer
	FANCD2	Breast cancer and Fanconi anemia
	KDR	Hemangioma, capillary infantile and hemangioma
	PDE11A	Pigmented nodular adrenocortical disease, primary, 2 and primary pigmented nodular adrenocortical disease
	RET	Multiple endocrine neoplasia iia and medullary thyroid carcinoma, familial
	RUNX1	Platelet disorder, familial, with associated myeloid malignancy and isolated delta-storage pool disease (among its related pathways are endometrial cancer and chronic myeloid leukemia)

Green	EPHB2	Prostate cancer/brain cancer susceptibility, somatic and prostate cancer
	NF1	Neurofibromatosis-Noonan syndrome and neurofibromatosis, type 1
	SDHC	Paragangliomas 3 and paraganglioma and gastric stromal sarcoma (among its related pathways are Alzheimer's disease and carbon metabolism)

Red	AXIN2	Oligodontia-colorectal cancer syndrome and colorectal cancer
	EHBP1	Prostate cancer, hereditary, 12 and prostate cancer
	EPHB2	Prostate cancer/brain cancer susceptibility, somatic and prostate cancer
	GLMN	Glomuvenous malformations and glomangioma
	GPC3	Simpson-Golabi-Behmel syndrome, type 1 and Wilms tumor susceptibility-5
	KDR	Hemangioma, capillary infantile and hemangioma
	KIT	Endometrial cancer in gastrointestinal stromal tumor
	MSMB	Prostate cancer, hereditary, 13 and prostate cancer
	MSR1	Prostate cancer and Barrett esophagus/esophageal adenocarcinoma
	PDGFRA	Gastrointestinal stromal tumor and hypereosinophilic syndrome, idiopathic, resistant to imatinib
	RUNX1	Platelet disorder, familial, with associated myeloid malignancy and isolated delta-storage pool disease (among its related pathways are endometrial cancer and chronic myeloid leukemia)
